# BRAT1 deficiency causes increased glucose metabolism and mitochondrial malfunction

**DOI:** 10.1186/1471-2407-14-548

**Published:** 2014-07-29

**Authors:** Eui Young So, Toru Ouchi

**Affiliations:** Department of Cancer Genetics, Roswell Park Cancer Institute, Elm and Carlton Streets, 14263 Buffalo, NY USA

**Keywords:** BRAT1, Glucose metabolism, Mitochondria, ROS

## Abstract

**Background:**

BRAT1 (BRCA1-associated ATM activator 1) interacts with both BRCA1, ATM and DNA-PKcs, and has been implicated in DNA damage responses. However, based on our previous results, it has been shown that BRAT1 may be involved in cell growth and apoptosis, besides DNA damage responses, implying that there are undiscovered functions for BRAT1.

**Methods:**

Using RNA interference against human BRAT1, we generated stable BRAT1 knockdown cancer cell lines of U2OS, Hela, and MDA-MA-231. We tested cell growth properties and *in vitro*/*in vivo* tumorigenic potentials of BRAT1 knockdown cells compared to control cells. To test if loss of BRAT1 induces metabolic abnormalities, we examined the rate of glycolysis, ATP production, and PDH activity in both BRAT1 knockdown and control cells. The role of BRAT1 in growth signaling was determined by the activation of Akt/Erk, and SC79, Akt activator was used for validation.

**Results:**

By taking advantage of BRAT1 knockdown cancer cell lines, we found that loss of BRAT1 expression significantly decreases cell proliferation and tumorigenecity both in vitro and in vivo. Cell migration was also remarkably lowered when BRAT1 was depleted. Interestingly, glucose uptake and production of mitochondrial ROS (reactive oxygen species) are highly increased in BRAT1 knockdown HeLa cells. Furthermore, both basal and induced activity of Akt and Erk kinases were suppressed in these cells, implicating abnormality in signaling cascades for cellular growth. Consequently, treatment of BRAT1 knockdown cells with Akt activator can improve their proliferation and reduces mitochondrial ROS concentration.

**Conclusions:**

These findings suggest novel roles of BRAT1 in cell proliferation and mitochondrial functions.

**Electronic supplementary material:**

The online version of this article (doi:10.1186/1471-2407-14-548) contains supplementary material, which is available to authorized users.

## Background

BRAT1 (BRCA1-associated ATM activator-1) was isolated as BRCA1 binding protein, interacting with the BRCT domain of BRCA1 [1]. Biochemical analysis indicated that pathogenic forms of the BRCT domain of BRCA1 protein (e.g. M1775R) do not bind to BRAT1, suggesting BRCA1/BRAT1 interaction is important for BRCA1’s tumor-suppressive functions. Mechanisms of sensing and repairing DNA lesions are well conserved among the species, and ATM, ATR and DNA-PK are essential for this mechanism [2]. Subsequent studies have shown that BRAT1 also binds to ATM and DNA-PKs, implicating the broad role of BRAT1 in DNA repair as well as in DNA damage response in general [1, 3, 4].

Previous studies have also illustrated BRAT1 acts as a regulator of cell growth and apoptosis. When BRAT1 was knocked down in mouse embryonic fibroblasts (MEFs) and human osteosarcoma cell (U2OS), a constitutive level of apoptosis was increased [1]. Interestingly, these studies have shown that ionizing radiation (IR) does not further induce apoptosis of these BRAT1 knockdown cells.

Recent genetic mapping and exome sequencing analysis identified that insertion mutations in the BRAT1 coding exon are pathogenic and cause lethal neonatal rigidity and multifocal seizure syndrome (RMFSL) [5, 6]. This disease is a lethal, neonatal, neurologic disorder characterized by episodic jerking, lack of psychomotor development, axial and limb rigidity, frequent multifocal seizures, and dysautonomia. Infants show poorly responsive focal jerks of the tongue, face and arms in a nearly continuous sequence throughout life. These results indicate the clinical relevance of BRAT1 pathways.

Mitochondria are critical organelles with important roles in cellular energy metabolism, which produces ATP via tricarboxylic acid (TCA) cycle and oxidative phosphorylation (OXPHOS) [7]. Also, mitochondria plays a key role in program cell death (apoptosis) as major site, where pro- and anti-apoptotic proteins interact and activates, so-called, mitochondria-dependent intrinsic apoptosis [8]. Mitochondrial failure by chemical or under disease condition induces increased reactive oxygen species (ROS) generation and mitochondrial membrane potential loss, leading to sequential apoptotic pathways, such as release of cytochrome c and activation of caspases [9]. Recent studies suggested that ROS generation and inhibition in mitochondrial functions are critical steps in chemical or knockdown-induced apoptosis of cancer cells [10–12]. In contrast, since Warburg discovered metabolic alterations in cancer cells (Warburg effect, the increase in aerobic glycolysis and the dependency on glycolytic pathway for ATP generation) [13, 14], a mitochondrial malfunction in respiration systems, due to mitochondrial DNA mutations/deletions, has been known as one of typical phenotypes in tumor tissues and cells [15, 16].

Recently, our previous studies showed the potential roles of BRAT1 not only in DNA damage responses, but also cell growth and apoptosis [1, 17]. In current study, we found that BRAT1 is involved in cellular growth properties including cell proliferation and tumor growth, and required for mitochondrial homeostasis, describing new roles of BRAT1 in cell growth and metabolism, and providing novel strategies for cancer treatment.

## Methods

### Cells and reagents

HeLa (human cervical carcinoma), U2OS (osteosarcoma), and MDA-MA-231 (human adenocarcinoma) cells were purchased from American Type Culture Collection (ATCC, Manassas, VA). All of these cells were cultured in DMEM media (Invitrogen, Carlsbad, CA) containing 10% fetal bovine serum (FBS, Invitrogen) and antibiotics. For starvation experiments, FBS were deprived for 24 h. Hydroxyurea (HU), Neocarzinostatin (NCS), and 2-Dexyl-D-glucose (2DG) were purchased from Sigma (St. Louis, MO). SC79, Akt activator was provided by Dr. Hongbo R. Luo (Harvard Medical School, Boston, MA). MitoTracker (mitochondrion-selective probe), MitoSOX (mitochondrial superoxide indicator), and CM-H2DCFDA (general oxidative stress indicator) were obtained from Invitrogen. JC-1 (mitochondrial membrane potential dye) was purchased from eBioscience (San Diego, CA).

### Plasmid and BRAT1 knockdown stable cell lines

Sure Silencing shRNA plasmids for human C7orf27 were purchased from SABiosciences (Valencia, CA). To avoid nonspecific targeting and increase efficiency, 4 independent target sequences and 1 nonspecific sequences (NC) were used as follows: #1: CCAGGACCCTGAGAGTTATGT, #2: TCTCTTCCTGAGGGACAAGAT, #3: GAGTTACTACCAGGGCTCTTT, #4: GCAGTTCCTCAGAGAGCTGTT, and NC: GGAATCTCATTCGATGCATAC. U2OS, HeLa, and MDA-MA-231 cells were transfected with shRNA plasmids using Lipofectamine 2000 transfection reagent (Invitrogen) according to manufacturer’s instruction. Cells were then cultured for 2 weeks in 3 μg/ml puromycin (Calbiochem, Billerica, MA) and single cell colonies were picked for analysis for BRAT1 expression by western blot.

### Immunoblotting and protein assays

Cells were treated for the indicated time, and then lysed in ice-cold lysis buffer (50 mM Tris–HCl (pH 7.6), 150 mM NaCl, 1 mM EDTA (pH 8.0), 20 mM NaF, 1 mM Na_3_VO_4_, 1% NP40, 0.5 mM dithiothreitol) in the presence of protease-inhibitor mix (leupeptin, aprotinin, and Phenylmethylsulfonyl fluoride, 10 μg/ml, respectively). After centrifugation (12000 g, 10 min), soluble supernatants were prepared and protein concentrations were calculated using the Bio-Rad protein assay kit. Total cell lysate (20 μg) was loaded and separated by 6.0% SDS polyacrylamide gels. Transfer to a PVDF membrane (Immobilon-P, Millipore) was done using semidry transfer method (Trans-Blot, Bio-Rad) in 25 mM Tris, 192 mM glycine, and 10% methanol for 1 h at 20 V. Membranes were blocked in 5% nonfat dried milk in Tris-buffered saline (TBS)/0.1% Tween 20 and incubated with primary antibodies and horseradish peroxidiseconjugated secondary antibodies (Santa Cruz Biotechnology, Santa Cruz, CA) followed by enhanced chemiluminescence detection. Primary antibodies used in this study were anti-Akt, anti-Erk (Santa Cruz Biotechnology), anti-BRAT1 (abcam, Cambridge, MA), anti-mTOR (Cell Signalling Technology, Danvers, MA). Also specific anti- phosphorylation antibodies were used against phospho Akt (Ser473, Thr308), phosphor-mTOR (Ser2448) and phosphor-Erk (Thr202/Tyr204) (Cell Signaling). Anti-actin antibody (Santa Cruz Biotechnology) was used to validate protein amount.

### Cell cycling and apoptosis analysis using flow cytometer

Both control and BRAT1 knockdown cells were exposed to vehicle (DMSO), or NCS (1 μg/ml) or HU (5 μM) for 24 h. Cell cycle arrest was assessed by ploidy analysis after DNA staining with propidium iodide using flow cytometer (FACSCalibur, BD Biosciences, Franklin Lakes, NJ) as previously described [18]. Apoptosis was determined by annexinV/PI double staining kits (BD Biosciences) according to manufacturer’s instruction. For experiment involving glucose starvation, cells were grown in DMEM with or without glucose for indicated days, and stained with PI. 2DG-induced apoptosis was determined compared with that in PBS-treated cells after 24 h treatment. The data were analyzed with CellQuestPro software (BD Biosciences).

### Wound healing and migration assay

Cells were treated with mitomycin C (30 μg/ml) for 30 min before a wound was made. The injury lines were created on 100% confluent monolayers of cells by scraping a gash using a micropipette tip. After being washing with PBS, cells were cultured in 10% complete DMEM for 46 h to be monitored wound healing. Photographs were taken at 22 h and 46 h under 40× magnifications using a SPOT Insight mosaic microscope camera (SPOT Imaging Solutions, Sterling Heights, MI) attached to Leica DM IRB microscope (Buffalo Grove, IL). For migration assay, control and BRAT1 knockdown MDA-MA-231 cells were suspended with 0.3 ml plain DMEM and then seed into 8.0 μm migration filters (BD FALCON) placed in 24-well plates. Complete DMEM medium 0.6 ml was added to the lower chamber. The plates were then incubated at 37°C for 16 h. Cells on the upper membrane surface were removed using a cotton tip, and migrated cells (on lower membrane surface) were fixed and stained by Diff-Quick stain kit (Siemens, Malvern, PA). The migration rate was determined by counting cells on lower side of membrane. Photographs were taken under 10× magnifications using Olympus DP70 digital camera (Center Valley, PA) attached to a Leica MZ 12 s microscope.

### Tumor formation in nude mice

Female athymic nude mice were purchased from Jackson lab (Bar Harbor, Maine), and housed in specific pathogen-free conditions. A total 2 × 10^6^ control and BRAT1 knockdown cells were subcutaneously injected into the flanks of nude mice. Mice were checked daily to examine tumor development, and tumor size was recorded at indicated days. Mice were euthanized and final tumors were isolated from mice, and then photographs were taken. These procedures were approved by the Institutional Animal Care and Use Committee (IACUC) of Roswell Park Cancer Institute.

### Cell proliferation assays

For direct cell number detection, cell were detached at indicated day by 0.1% Trypsin/EDTA solution (Invitrogen), and washed with PBS. Cell suspensions were mixed with an equal volume of 0.4% trypan blue (VWR, Radnor, PA), and viable cells (trypan blue negative cells) were counted. In some experiments, cell proliferation/viability was measured by an MTT assay (BMR Service, Buffalo, NY) according to manufacturer’s instruction. In brief, cell medium was aspirated and then 0.3 ml MTT working solution was added into 24 well. After 30 min incubation at 37oC, MTT solution was aspirated, and cells were incubated with 0.3 ml DMSO for 2 min. The DMSO extracts were transferred to a 96-well plate and absorbance was measured with micro-plate reader at a wavelength of 540 nm.

### ROS detection and measurement of mitochondrial membrane potential

For measurement of mitochondrial ROS, cells were cultured in complete DMEM containing 5 μM Mitosox for 10 min at 37°C, protected from light. Cells were washed three times with warm PBS, and then mounted with mounting medium with DAPI (Vector lab, Burlingame, CA). Fluorescent images were captured using Nikon TE2000-E inverted microscope equipped with a Roper CoolSnap HQ CCD camera (Melville, NY, USA). For detection of cellular ROS, cells were incubated with 5 μM CM-H2DCFDA for 1 h at 37°C, and then subjected to fluorescence microscopy. For quantitative assay, cells were detached by 0.1 trypsin/EDTA solution after incubation in Mitosox or CM-H2DCFDA working solution. Cell suspensions were analyzed by flow cytometry. To determine mitochondrial membrane potential, cells were stained with JC-1(2.5 μg/ml) for 10 min at room temperature and then analyzed by flow cytometry.

### Determination of Pyruvate dehydrogenase (PDH) activity and measurement of mitochondrial and intracellular ATP

PDH activity of control and BRAT1 knockdown cells was analyzed by Pyruvate Dehydrogenase assay kit (BMR Service). Membrane fraction was collected from cell lysates and re-suspended for assay. PDH activity was measured as O.D at 492 nm using microplate reader. Protein assay was performed to determine sample protein concentration before analysis. To measure of mitochondrial ATP, mitochondria were isolated by mitochondria isolation kit for cultured cells (Pierce Biotechnology, Rockford, IL). Total cell lysate for intracellular ATP was prepared by adding sterile water into wells. Mitochondrial and total ATP level were detected using ATP assay kit (BMR Service) according to manufacturer’s instruction. Luminescence was measured by Veritas microplate luminometer (Promega, Madison, WI) and ATP concentration of each sample was normalized to the protein concentration.

### Measurement of glucose consumption and lactate accumulation

Glucose assay kit and L-lactate assay kit (BMR service) were used to measure concentration of glucose and lactate in media from control and BRAT1 knockdown cultures. Culture media were prepared at indicated days and glucose and lactate levels were measured according to manufacturer’s instructions. Absorbance was measure at 492 nm and water (glucose) and DMSO (lactate) were used to detect base lines.

### Statistical analysis

Data are expressed as mean values ± standard deviation (SD); *p* values were calculated with an unpaired two-tailed Student’s *t*-*test*.

## Results

### BRAT1 expression is required for optimal proliferation and viability

**Figure 1:**
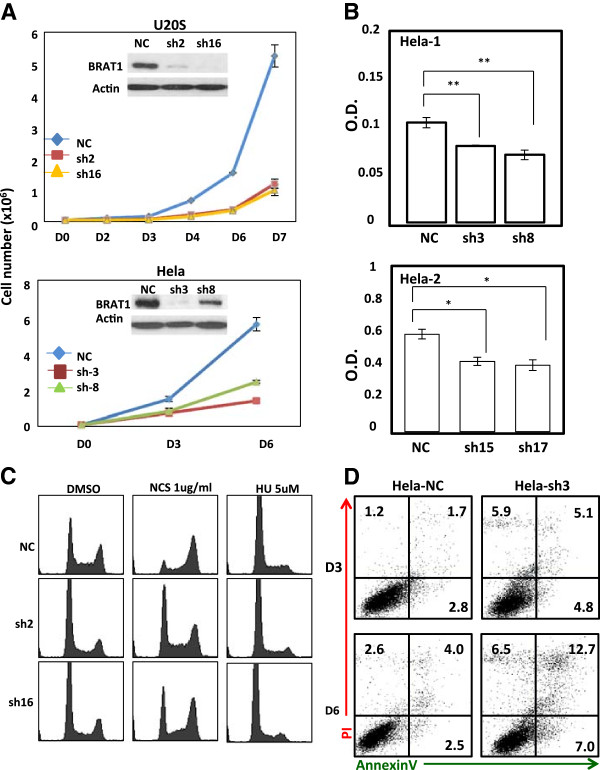
**BRAT1 expression is required for optimal proliferation and viability. (A)** NC (nonspecific shRNA) and Sh (selected BRAT1 knockdown cells) were selected and cloned from U2OS and HeLa parental cells after transfection with 4 different shRNA against BRAT1 mRNA. The expression of BRAT1 was confirmed by immunoblot (inserts). Actin protein was used as internal control. The number of live cells (trypan blue negative) was directly counted at indicated days. **(B)** 4 different BRAT1 knockdown HeLa cells (sh3, sh8, sh15, and sh17) were cultured for 3 days (upper panel) and indicated days (bottom panel), then cell proliferation was measured using the MTT assay. **(C)** Both control and knockdown U2OS cells were treated with NCS (1 μg/ml) or hydroxyurea (HU, 5 μM), then cultured for 24 h. Cells were fixed and stained with propidium iodide (PI). DNA profile was analyzed by a flow cytometry. **(D)** Both control and BRAT1 knockdown cells were cultured for indicated times without changing media, and then subjected to apoptosis analysis using AnnexinV/PI double stain. Apoptosis and necrosis were expressed by percentage from total cells in dot plot graphs. Data are mean of three independent experiments. **Student’s *t*-test: p < 0.01.

To detail the role of BRAT1 in cell proliferation, BRAT1 expression was stably knocked down in two different human cancer cells, U2OS (human osteosarcoma) cell line and HeLa (human cervical carcinoma) cell line, using BRAT1-targeted shRNA plasmids. Levels of BRAT1 were determined by immunoblot analysis. Sh2, Sh16 clones for U2OS cells and Sh3, Sh8 for HeLa cells showed much lowered expression of BRAT1 among the stable clones isolated and they were further studied for functional analysis of the protein (Figure 1A).

First, we studied the effect of BRAT1 silencing on cell growth by measuring cell number (Figure 1A) and the MTT (3-(4,5-dimethylthiazol-2-yl)-2,5-diphenyltetrazolium bromide, a yellow tetrazole) assay (Figure 1B). These experiments show that BRAT1 knockdown in both U2OS and HeLa cell lines results in extensive growth retardation. Next, we tested cell cycle profile by DNA staining with propidium iodide (PI), followed by flow cytometry analysis. We found that BRAT1 knockdown U2OS cells showed lower S-phase population (15.6 ± 2.7% in U2OS Sh2 and 16.2 ± 2.3% in U2OS Sh16) than control cells (30.2 ± 0.3%) (Figure 1C). When treated with neocarzinostatin (NCS, radio-mimetic chemical, 1 μg/ml), accumulation in G2/M-phases was observed in control U2OS cells (59.3 ± 5.9%), however this NCS-induced G2/M-arrest was abrogated in U2OS Sh2 and Sh16 cells (33.27 ± 0.5 and 42.9 ± 2.2% respectively), indicating that BRAT1 is involved in G2/M checkpoint under conditions of DNA damage as shown in our previous report [1]. Interestingly, U2OS Sh2 and Sh16 cells showed G1 arrest (10.3 ± 2.8 and 6.1 ± 1.0%, respectively) to the similar degree with that of control U2OS cells (7.8 ± 1.6%), when treated with hydroxyurea (Hu, 5 μM), suggesting that BRAT1 is not essential for HU-induced G1 checkpoint.

We next studied whether decrease in BRAT1 expression causes apoptosis. HeLa Sh3 cells were maintained without changing media and apoptosis was determined by Annexin V staining, followed by FACS analysis. We found that HeLa Sh3 cells showed increase in apoptosis (Annexin A^High^/PI^Low^) and necrosis (Annexin V^Low^/PI^High^) when cell culture is maintained for 3 days (D3) to 6 days (D6) compared to control cells (Figure 1D). These results suggest that BRAT1 is required to maintain cell viability.

### Loss of BRAT1 causes reduced cell migration and tumorigenesis

**Figure 2:**
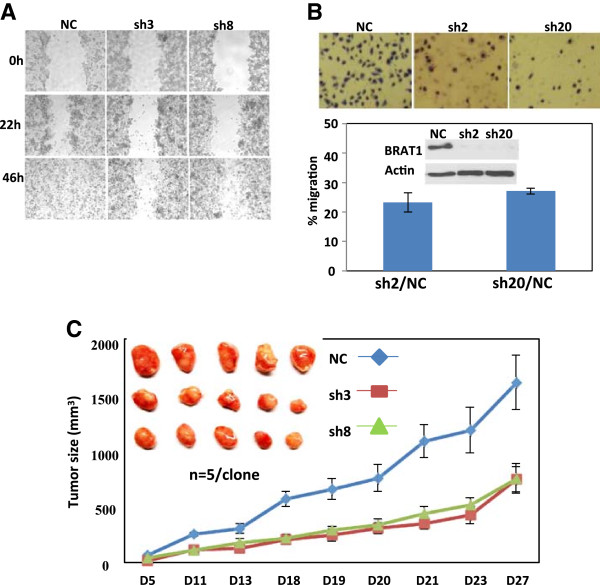
**Loss of BRAT1 induces morphological changes and in vivo tumor growth. (A)** The injury lines were made on confluent monolayers of control (NC) and BRAT1 knockdown (sh3 and sh8) HeLa cells. Wound healing potential was analyzed under a light microscope (×40) at indicated time points. **(B)** Both control (NC) and BRAT1 knockdown (sh2 and sh20) MDA-MA-231 cells were seeded onto migration chamber and infiltrated cells were stained with 0.1% crystal violet, counted and then quantified (bottom). Percentage of migration was expressed as ratio of migrating cells numbers of knockdown cells to control cells. The expression of BRAT1 was confirmed by immunoblot (inserts) Actin protein was used as internal control. **(C)** Control and BRAT1 knockdown HeLa cells were injected into female nude mice as described in M&M. Tumor size was measured at indicated days and then sacrificed at day 27. Tumors were isolated from individual mouse and photographs were taken. Data were representative of three independent experiments.

Increased cell migration and tumor formation are key characteristics of cancer cells. To further characterize the BRAT1-knockdown cells, we performed wound healing and migration assay. Both control (NC) and HeLa Sh3 and Sh8 cells were pretreated with mitomycin C before making injury lines to exclude the effect by proliferation. As shown in Figure 2A, wound healing activity of BRAT1 knockdown cells was severely impaired. Roles of BRAT1 in cell migration were studied with a migration chamber (Figure 2B). Control and BRAT1 knockdown MDA-MA-231 (231), human breast cancer cells, were used for this assay, since MDA-MA-231 cells have been frequently used for cell migration and penetration assay using matrigel [19, 20]. 231 cells were stably transfected with nonspecific shRNA or 4 different BRAT1shRNA, and then antibiotic-resistant clones were selected after 2 weeks as described. Knockdown of BRAT1 protein in these stable cells was confirmed by immunoblot (insert of Figure 2B). We found that 231 Sh2 and 231 Sh20 cells showed significantly decreased mobility, compared with control cells, which was determined by staining cells that infiltrated the membrane. Quantified analysis showed that migration of BRAT1 knockdown cells was more than 3 fold lower than that of control cells.

Next, we examined the tumorigenicity of BRAT1 knockdown cells in vivo by xenograft assay using HeLa Sh3 and Sh8 cells. Control, HeLa Sh3 and HeLa Sh8 cells were transplanted into nude mice (2 × 10^6^ cell/mouse), and size of the tumors was measured on day 5, 11, 13, 18, 19, 20, 21, 23, and 27. As shown in Figure 2C, tumor size of BRAT1 knockdown cells was almost half of control HeLa cells throughout the time course, indicating that BRAT1 regulates tumor cell growth. Together, these results indicate that BRAT1 is involved in tumor cell growth, tumorigenesis and cell motility.

### The rate of glycolysis and dependency on glucose are increased in BRAT1 knockdown cells

**Figure 3:**
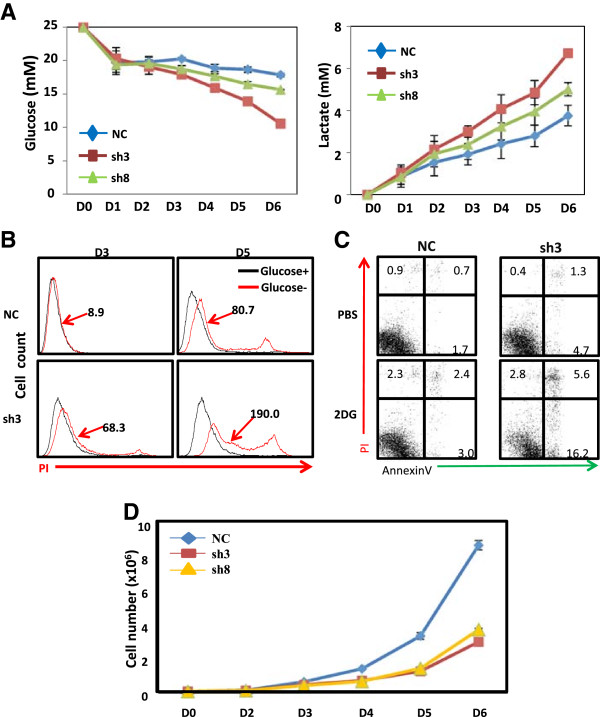
**The rate of glycolysis and dependency on glucose are increased in BRAT1 knockdown cells. (A)** Culture media were harvested from control or BRAT1 knockdown cell cultures at indicated day and then glucose consumption (left) and lactate accumulation (right) were analyzed. **(B)** Control and BRAT1 knockdown cells were cultured with (glucose +) or without glucose (glucose -). Cells were harvested at indicated day and stained with PI without fixation. PI positive cells were detected as apoptotic/necrotic cells. Data were expressed as mean fluorescence intensity (ΔMFI = MFI of PI stained cells – MFI of unstained cells). **(C)** Cells were treated with or without 2DG (5 mM) for 24 h and then cellular apoptosis was detected with AnnexinV/PI double staining by flow cytometry. Flow cytometry data were representatives of two different experiments. **(D)** Culture media were daily changed and the number of living cells was counted after trypton blue staining.

Increased glycolysis is one of the most prominent metabolic alterations in cancer cells [13] and this metabolic alteration increases aerobic glycolysis and dependency on the cytoplasmic glycolytic pathway for ATP generation, instead of mitochondrial TCA cycle [16]. We observed rapid changes of acidity of cell culture media of BRAT1 knockdown cells compared to control cells (see Additional file 1: Figure S1). Acidic pH in culture media implied that rate of glycolysis is increased in BRAT1 knockdown cells. To examine whether BRAT1 knockdown results in the change of glucose metabolism, we studied the rate of glucose consumption and lactate formation in both control and BRAT1 knockdown cells (Figure 3A). Increase in glucose consumption was measured daily by concentration of glucose in culture media. Compared to control cells, glucose concentration in culture media of HeLa Sh3 and Sh8 cells was lower than that of control cells, indicating that knockdown of BRAT1 results in higher glucose consumption. Increase in glucose metabolism was confirmed by lactate concentration in culture media. Thus, we found that HeLa Sh3 and Sh8 cells produce more lactate than control cells (Figure 3A, right).These results suggest that BRAT1 knockdown cells require more glucose for their growth. We further analyzed glucose metabolism in BRAT1 knockdown cells by maintaining cells with or without glucose in cell culture media, and their apoptosis was measured on day 3 and day 5 (Figure 3B). Apoptosis was quantified by mean fluorescence intensity (MFI). On day 3, control HeLa cells showed slight increase in apoptosis in glucose (−) media, but HeLa Sh3 was more sensitive to glucose deprivation. Increased sensitivity to glucose deprivation was more obvious on day 5, when, compared to control HeLa cells, HeLa Sh-3 cells showed much higher apoptosis.

2DG (2-deoxy-D-glucose) is a chemical analogous to glucose, which inhibits glucose metabolism by causing glucose starvation [21–23]. Increased sensitivity of BRAT1 knockdown cells to glucose deprivation was further studied to maintain those cells in the presence of 2DG (5 mM). Early to late apoptosis was determined by Annexin V staining using flow cytometry. As shown in Figure 3C, 2DG treatment induced apoptosis of control HeLa cells. Apoptosis of HeLa Sh-3 cells was constitutively high, and it was further increased when cells were treated with 2DG. These results support a notion that BRAT1 knockdown cells are more sensitive to glucose deprivation.

### Loss of BRAT1 induces mitochondrial malfunctions

Several groups have suggested that DNA damage response protein ATM is required for mitochondrial function, which ATM plays direct roles in modulating mitochondrial homeostasis and ATM deficiency induces mitochondrial dysfunction and increase mitochondrial ROS [24, 25]. As we reported previously, BRAT1 is essential for the activation of ATM and DNA-PKcs [1, 3, 4]. Thus, we investigated whether BRAT1 is required for mitochondrial functions.

**Figure 4:**
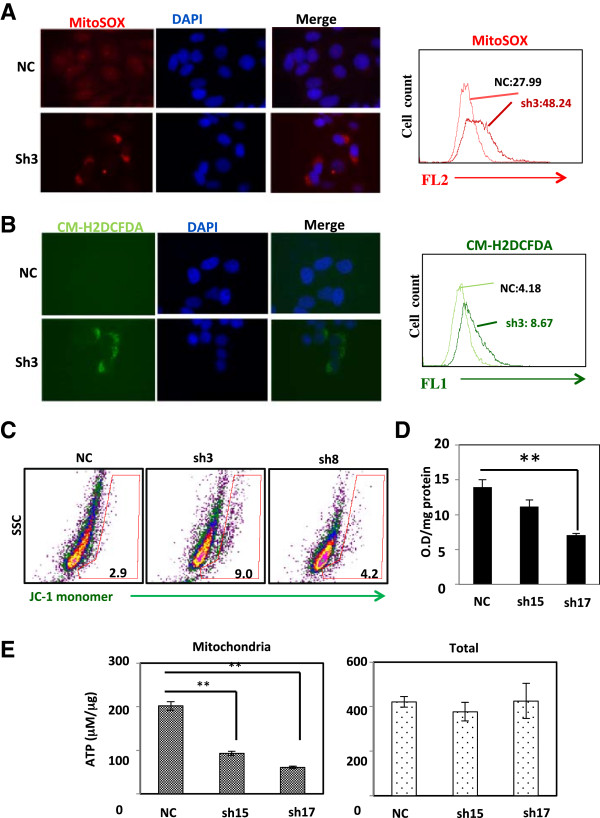
**BRAT1 is required for mitochondrial functions.** Mitochondrial **(A)** and cellular **(B)** ROS levels in control (NC) and BRAT1 knockdown (sh3) HeLa cells were detected by mitosox (red), CM-H2DCFDA (green), and DAPI using fluorescent microscopy. Quantitative flow cytometry data (right) were expressed as ΔMFI. **(C)** Control and BRAT1 knockdown HeLa cells were stained with JC-1 dye at 2.5 μg/ml for 10 min and harvested for flow cytometry. The percentage of JC-1 monomer positive cells was expressed in gates. **(D)** Total lysate was isolated from control (NC) and BRAT1 knockdown (sh15 and sh17) HeLa cells and then subjected to array for PDH activity. The activity was expressed as O.D. per mg protein at 492 nm using a microplate reader. **(E)** Mitochondria were isolated from control (NC) and BRAT1 knockdown (sh15 and sh17) HeLa cells, lysed and then ATP concentration were measured as μM/μg proteins (left). Total cell extracts from same cells were also analyzed for total cellular ATP (right). Data were representative of three independent experiments. **Student’s *t*-test: p < 0.01.

First, we found that distribution of mitochondria in HeLa knockdown clone is different from that of control cells when cells are stained with dye that localizes mitochondria. In control cells, mitochondria localization is dispersed in the cytoplasm, but it is more condensed in BRAT1 knockdown cells (see Additional file 2: Figure S2). Because proper mitochondrial distribution is essential for mitochondrial functions, such as ATP delivery and calcium regulation [26], this change in mitochondrial distribution suggests that BRAT1 is involved in mitochondria homeostasis. Supporting this model, we found that BRAT1 localizes in both nuclear and cytoplasm [1]. Thus, cytoplasmic BRAT1 might be involved in this mitochondria regulation. Based on preliminary data, we tested the production of superoxide by mitochondria with fluorescence microscopy using the MitoSOX reagent. It permeates live cells where it selectively targets mitochondria, and is rapidly oxidized by superoxide but not by other reactive oxygen species (ROS) and reactive nitrogen species (RNS). As shown in Figure 4A, production of mitochondrial superoxide is significantly increased in HeLa Sh3 cells, compared to the control cells. Intensity of fluorescence was quantified by MFI.

Next, we monitored the generation processes of reactive oxygen species (ROS) using the luminescence analysis of 2’,7’-dichlorfluorescein-diacetate (DCFH-DA), which has been broadly used as a compound to detect and quantify intracellular produced ROS. Quantified fluorescent signal analysis determined by MFI indicated that HeLa Sh3 cells contain much higher levels of ROS compared to the parental HeLa cells (Figure 4B).The membrane-permeant JC-1 (5,5′,6,6′-tetrachloro-1,1′,3,3′-tetraethylbenzimidazolocarbo-cyanine iodide) dye is widely used to monitor mitochondrial health, and can be used as an indicator of mitochondrial membrane potential in a variety of cell types. The dissipation of the mitochondrial electrochemical potential gradient is known as an early event in apoptosis. In normal cells, due to the electrochemical potential gradient, the JC-1 concentrates in the mitochondrial matrix. Any event that dissipates the mitochondrial membrane potential prevents the accumulation of the JC-1 dye in the mitochondria and thus, the dye is dispersed throughout the entire cell leading to a shift to green fluorescence (JC-1 monomers). When we tested JC-1 signal of control, Sh3 and Sh8 HeLa cells, we observed increase in JC-1 monomeric signals in both HeLa Sh3 and Sh8 cells, illustrating mitochondrial dysfunction in HeLa Sh3 and Sh8 cells (Figure 4C).

**Figure 5:**
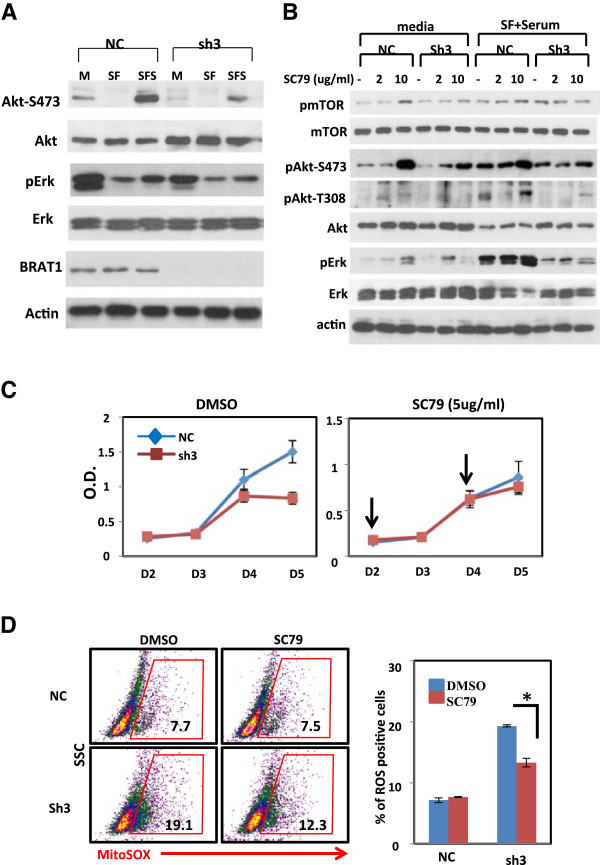
**Loss of BRAT1 leads to inhibition of Akt activity and Akt activation by SC79 partially restores BRAT1 knockdown cells. (A)** Control (NC) and BRAT1 knockdown (sh3) HeLa cells were cultured in DMEM media with 10% serum (M). To examine serum-induced activation, cells were cultured in DMEM media without serum (SF and SFS) for 24 h and then continuously cultured with 10% serum (SFS) or without serum (SF) for 1 h. Protein extracts were blotted using indicated antibodies for phospho- or whole proteins. Actin protein was used as internal control. **(B)** Cells were cultured with (media) or starved for 24 h and then serum was added into these media. SC79 (5 μg/ml) were treated at 2, 10 μg/ml for 30 min and then total extracts were analyzed for phosphorylation and expression of indicated protein by immunoblotting. **(C)** Cells were cultured with or without SC79 for indicated time and then cell proliferation was measured using MTT assay. More SC79 was added at D2 and D4 (down arrow). **(D)** Cells were cultured with or without SC79 (5 μg/ml) for 24 h and stained with mitosox for 10 min. Flow cytometry was conducted to detect mitosox positive cells (gated) from control and BRAT1 knockdown cells. Quantified flow cytometry data (left) were representative of two independent experiments (right). *Student’s *t*-test: p < 0.05.

Taken together, these results suggest that BRAT1 depletion results in mitochondrial malfunction, leading to increased metabolism of glucose consumption. It is assumed that these cells are more sensitive to glucose deprivation.

Pyruvate dehydrogenase (PDH) transforms pyruvate into acetyl-CoA, contributing to linking the glycolysis metabolic pathway to the tricarboxylic acid (TCA) cycle [27]. ATP production from mitochondria is one of criteria to evaluate mitochondrial function [28]. In this assay, endogenous PDH reduces tetrazolium salt, INT (2-p-iodophenyl-3-nitrophenyl-5-phenyl tetrazolium chloride) to INT-formazan in a NADH-coupled reaction. The intensity of the red color formed is increased in the presence of increased PDH activity. As shown in Figure 4D, PDH activity was reduced in BRAT1 knockdown cells. Next, we tested if BRAT1 is involved in mitochondrial or cytoplasmic ATP production. In this assay, enzyme luciferase catalyzes the oxidation of luciferin, in ATP-dependent manner, which can be measured by a luminometer. As shown in Figure 4E, the level of mitochondrial ATP was significantly lower in BRAT1 knockdown cells compared with control cells, but the total levels of cellular ATP were not significantly different. These results indicate that ATP production from mitochondria is decreased in BRAT1 knockdown cells, suggesting that BRAT1 cells shift their energy source toward glycolysis to generate their ATP supply. Taken together, present data demonstrate that BRAT1 plays a critical role in regulating mitochondrial functions.

### BRAT1 is required for constitutive Akt activation, and Akt activation by SC79 partially restores BRAT1 knockdown cells

It has been well documented that PI3K/Akt and extracellular signal-regulated kinase (Erk) signaling cascades regulate a wide variety of cellular processes, such as cell proliferation, differentiation, survival, cell transformation and metastasis of tumor cells [29, 30]. Further, Akt activation stimulates glucose consumption in transformed cells, and constitutive active Akt-expressing cells were more susceptible to glucose deprivation than Akt-deficient cells [31]. Also recent works suggested that mitochondrial stress leads to increased expression, activation, and nuclear localization of Akt [32]. Together, these works suggested that glucose metabolism inhibits mitochondrial oxidation and suppresses apoptosis and increase proliferation in cancer cells by Akt-mediated signal. However, mitochondrial failure without increase in glucose metabolism suppresses cell growth and increase apoptotic phenotypes of cancer cells [33, 34].Because our data showed that mitochondria function is impaired in BRAT1 knockdown cells, we studied whether growth promoting pathways are activated in those cells. The expression of Akt, Erk and their phosphorylation status were assessed by western blotting. As shown in Figure 5A, phosphorylation of both Akt and Erk decreased in BRAT1 knockdown cells. Serum-induced activation of these kinases is significantly reduced in knockdown cells. Given the low migration and tumorigenesis of BRAT1 knockdown cells, these results suggest that phosphorylated Akt is indicative of reduced cell proliferation. We continue to study the mechanism of lowered Akt phosphorylation of BRAT1 knockdown cells, even though these cells consume more glucose than the control cells.

Recently, Luo’s laboratory developed a novel Akt activator (SC79) which augments neuronal survival in mouse model for ischemic stroke [35]. SC79 directly enhanced Akt phosphorylation of all Akt isoforms and increases Akt activity in multiple cell types, including HeLa, HL60, HEK293, NB4 and HsSulton cells. When HeLa Sh3 cells were treated with SC79, Akt’s phosphorylation at Ser473 and Thr308 were induced, although it was slightly weaker than that of control cells (Figure 5B). We explored the effect of SC79 on cell proliferation of BRAT1 knockdown cells using MTT assay. As shown in Figure 5C, SC79 treatment restored proliferation of BRAT1 knockdown cells to the similar degree of control cells. We also found that SC79 reduces the production of superoxide in mitochondria that was detected by MitoSox positive cells (Figure 5D). These data clearly indicate that the loss of BRAT1 inhibits growth signaling cascades mediated by Akt pathways.

## Discussion

It has been implicated that BRAT1 might be a regulator for ATM and DNA-PK activation in response to DNA damage induced by ionizing radiation (IR) or chemicals [4]. Interestingly, silencing of BRAT1 increased constitutive apoptosis and reduced cell growth. In this study, we determined a role for BRAT1 in proliferation and mitochondrial functions. After confirming suppressed BRAT1 expression, we found reduced BRAT1 expression in multiple cell lines induces growth retardation, increased apoptosis, and reduced tumor growth in vivo (Figures 1 and 2). This data suggests that BRAT1 has play a role in tumorigenesis, but further studies will be needed to identify BRAT1 role for whole tumor progress, including metastasis of specific tumor models.

It was interesting that BRAT1 knockdown cells used inefficient glucose, leading to fast reduction in pH. We first found acidic extracellular pH through phenol red color (Figure S1). Although BRAT1 knockdown decreased cell proliferation, higher glycolysis and increased lactate accumulation were observed in BRAT1 knockdown culture media (Figure 3A), suggesting that glucose metabolism was modulated in correlated with reduced expression of BRAT1. To support this notion, we showed the glucose deprivation and blocking glycolysis by 2DG induce more severe apoptosis in BRAT1 knockdown cells than in control hela cell (Figure 3B and C). However, our data suggests that increased dependency on glucose is not direct reason of growth retardation and constitutive apoptosis as shown in BRAT1 knockdown cells (Figure 3D).

Several possibilities could be suggested why glucose consumption might be increased in BRAT1 knockdown cells. These mechanisms may include mitochondrial malfunction and oncogenic signaling, such Ras and Akt [31, 36]. Mitochondrial oxidative phosphorylation and cytoplasmic glycolysis are two main metabolic pathways by which ATP is generated for energy supply [37]. Mitochondrial malfunction has been implicated to be responsible for increased glycolysis [16]. Impaired mitochondrial function also causes pyruvate accumulation in cancer cells [38]. Therefore, we reasoned that high glucose consumption might be due to mitochondrial malfunction. Data in Figure 4 shows that 4 different analyses revealed aberrant mitochondrial functions and metabolic pathways. In other words, elevated level of ROS, lower mitochondrial membrane potential, impaired PDH activity, and decreased production of ATP from mitochondria in BRAT1 knockdown cells clearly describe that BRAT1 has play a critical role in mitochondrial functions.

It was reported that mitochondrial respiration defects lead to activation of Akt survival pathway through a mechanism mediated by NADH, describing how metabolic alteration in cancer cells gain a survival advantage [15]. Also recent work suggested that mitochondrial stress leads to increased expression, activation, and nuclear localization of Akt [36]. Because our data showed a series of mitochondrial-originated stresses, we expected that Akt pathway might be constitutively activated in BRAT1 knockdown cells. However, both basal and serum-induced activation of Akt were reduced in BRAT1 knockdown cells (Figure 5A and 5B), suggesting that knockdown-induced cellular and mitochondrial stress is not able to activate Akt. Further, Erk phosphorylation was also decreased in these cells. Akt and Ekr-mediated signaling pathways are critical steps for a wide variety of cellular processes, including cell survival, growth, proliferation, metabolism and migration [29, 30]. Thus, we couldn’t detect any stress-induced Akt or Erk activation in BRAT1 knockdown cells, instead, results implicates that BRAT1is involved in Akt/Erk-mediated growth regulation. SC79 can enhance Akt-PDK1 interaction, leading to enforced phosphorylation at Thr308 and Ser473 of Akt [35]. Using SC79, we confirmed Akt activation can moderately restore cell growth and ROS level (Figure 5C and 5D). Although SC79 treatment was able to induce Akt phosphorylation in knockdown cells, the level of phosphorylation was less than control, suggesting that the upstream of Akt might be impaired by suppressed expression of BRAT1. It is interesting further study to investigate whether BRAT1 is involved in PI3K/mTOR/Akt signaling pathway, leading to optimal growth and keeping metabolic homeostasis of mitochondria.

So far, BRAT1 has been the only DNA damage response protein, which regulates reaction stability of ATM/DNA-PK, leading to genomic stability after DNA damaging stress. However, this protein seems to account for proliferation and cellular metabolism in correlated with mitochondrial functions. Genetic investigation of patients with mutated BRAT1 suggests that BRAT1 plays a role on neuronal development [5]. They found deletion mutant of BRAT1 in patient, however it remains to be clear how this mutation affect disease development.

## Conclusions

In the current studies, we demonstrate that loss of BRAT1 induces mitochondrial malfunctions, suppresses growth signaling, and increase steady-state levels apoptosis. We also show that growth retardation in BRAT1 knockdown cells is due to mitochondrial malfunction that causes increased ROS and decreased ATP production. It has been illustrated that BRAT1 regulates activation of ATM and DNA-PK under conditions of DNA damaging stress. Present results indicate that BRAT1 is also involved in cell proliferation and mitochondrial metabolism. Because ATM-deficient cells showed mitochondrial dysfunction [29], abnormal cellular differentiation/development [39], and impaired Akt activation [40], our results further support the model that BRAT1 determines the phenotypes caused by ATM deficiency.

## Electronic supplementary material

Additional file 1: Figure S1: Remarkable PH changes in media from BRAT1 knockdown cultures compared to that from control culture. PH of media from control (NC) and BRAT1 knockdown (sh3 and sh8) HeLa cell cultures were directly recorded by PH meter (Mettler-Toledo, LLC, OH) at day 2 and day 5 after seeding (2× 10^5^/6 cm culture dish). (PDF 748 KB)

Additional file 2: Figure S2: Loss of BRAT1 induces morphological changes. (A) Both control (NC) and knockdown (sh3) HeLa cells were seeded onto 6 cm plates and cultured for 24 h. Cells were treated with MitoTracker (MT) for 10 min before fixation, then morphological features were analyzed. (B) Both control (NC) and BRAT1 knockdown (sh20) MDA-MA-231 cells were examined with a bright field inverted microscope (Nikon). (PDF 1 MB)

## Abbreviations

BRCA1: Breast Cancer 1
ATM: Ataxia Telangiectasia Mutated
BRAT1: BRCA1-associated ATM activator 1
DNA-PKcs: DNA-activated Protein Kinase, catalytic subunit
ROS: Reactive oxygen species
RNS: Reactive Nitrogen Species
MEFs: Mouse Embryonic Fibroblasts
IR: Ionizing Radiation
RMFSL: Rigidity and Multifocal Seizure Syndrome
TCA: Tricarboxylic Acid
OXPHOS: Oxidative Phosphorylation
MTT: 3-(4,5-dimethylthiazol-2-yl)-2,5-diphenyltetrazolium bromide, a yellow tetrazole
NCS: Neocarzinostatin
Hu: Hydroxyurea
MFI: Mean Fluorescence Intensity
2DG: 2-deoxy-D-glucose
DCFH-DA: 2’,7’-dichlorfluorescein-diacetate
JC-1: 5,5′,6,6′-tetrachloro-1,1′,3,3′-tetraethylbenzimidazolocarbocyanine iodide
PDH: Pyruvate Dehydrogenase.
