# Supplementary figures and images for: BRAT1 deficiency causes increased glucose metabolism and mitochondrial malfunction
Source: BMC Cancer. 2014 Jul 29;14:548. doi: 10.1186/1471-2407-14-548 (PMC4129107; doi:10.1186/1471-2407-14-548)

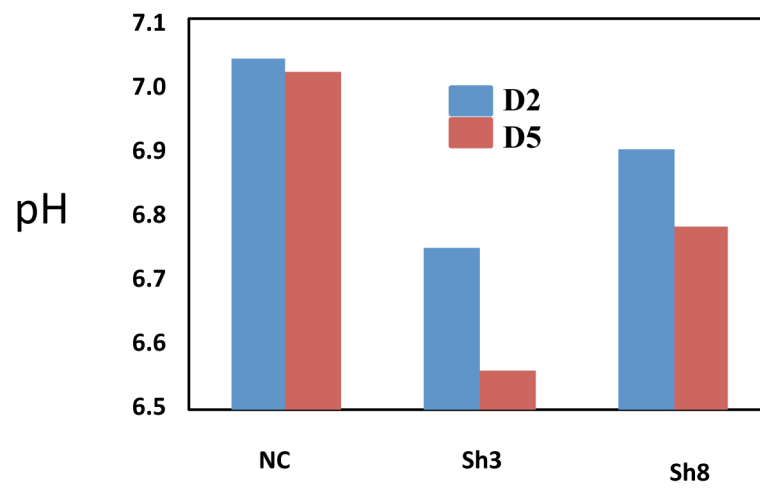

**Supplement Figure 2**

Supplement: Supplementary file 1 — Additional file 1: Figure S1: Remarkable PH changes in media from BRAT1 knockdown cultures compared to that from control culture. PH of media from control (NC) and BRAT1 knockdown (sh3 and sh8) HeLa cell cultures were directly recorded by PH meter (Mettler-Toledo, LLC, OH) at day 2 and day 5 after seeding (2× 105/6 cm culture dish). (PDF 748 KB) [file 12885_2014_4739_MOESM1_ESM.pdf]

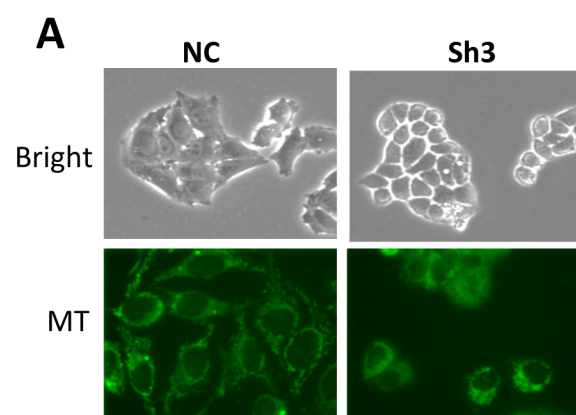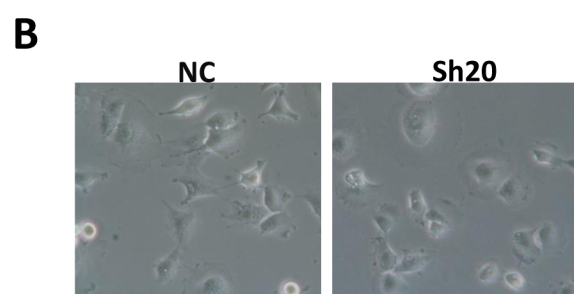

**Supplement Figure 3**

Supplement: Supplementary file 2 — Additional file 2: Figure S2: Loss of BRAT1 induces morphological changes. (A) Both control (NC) and knockdown (sh3) HeLa cells were seeded onto 6 cm plates and cultured for 24 h. Cells were treated with MitoTracker (MT) for 10 min before fixation, then morphological features were analyzed. (B) Both control (NC) and BRAT1 knockdown (sh20) MDA-MA-231 cells were examined with a bright field inverted microscope (Nikon). (PDF 1 MB) [file 12885_2014_4739_MOESM2_ESM.pdf]
